# Neurology

**Published:** 1904-09-24

**Authors:** 


### NEUROLOGY.

(*Continued from page* 433.)

**Insomnia.**—A practical paper on the treatment of this affection has been written by Gordon.[4] With the incidence of sleep it should be remembered that certain physiological phenomena occur : there is a contraction of the cerebral vascular system, and a corresponding dilatation of the vessels of the periphery throughout the body. There is also marked slowing of the pulse-rate and diminution in the force of the heart's action. The respiration is deepened and slowed, with a corresponding retardation in all the metabolic and secretory processes. The causes of sleeplessness are manifold. Many cases of mania and neurasthenia are preceded by insomnia ; other causes are alcoholic and various excesses, worry, grief, anxiety, over-fatigue. The hygienic treatment, which in many cases is of far more value than the medicinal, consists in the use of various hydro-therapeutic measures—the warm foot-bath, cold pack, sitz-bath, the ingestion of hot drinks, and regulation of the mode of life. The medicinal treatment consists in the regulation of the alimentary tract and the exhibition of medicine. The warm full bath given just before retiring, followed by a hot drink and a hypnotic in moderate dose, is of great value. The water should be at the temperature of 98°, and for half an hour, and the head should be enveloped, above the eyes, in a towel wrung out of cold water. This should be followed by a hot glass of milk and the hypnotic. The cold wet pack is equally efficacious. The sensation of cold disappears in a few minutes, and dilatation of the skin vessels follows. Even excited sleepless patients will often go to sleep in the pack. The sheet should be wrung out of water at a temperature of 70°, and outside this blankets should be wrapped round the patient. Warm sitz baths and foot-baths also exert a distinctly sedative and anodyne effect. These are simple hygienic measures of great value, though often neglected. The principle underlying them is the depletion of the cerebral circulation they cause. Of drugs, paraldehyde is one of the best ; it may be given in the following mixture : paraldehyde two parts, whisky one part, syrup of orange one part—to be shaken just before administration. The dose of the paraldehyde is two drachms. In alcoholics particularly this remedy is [illegible] superior to the usual chloral and bromide mixture ; it has no depressing after effects, does not lock up the secretions and does not affect the heart. Trional in 15 to 30 gram doses acts well if given one or two hours before bed-time. Sulphonal often has a depressing and unpleasant after-effect and a tendency to check secretion. Insomnia caused by overfatigue should not be treated by hypnotics, but by the exhibition of a mild stimulant such as strychnine or a glass of ale or porter. The stimulating effect of the half bath or a simple cold dip often acts like magic in these cases.

**Giddiness.**—In a clinical lecture Smith[5] said that giddiness is of two kinds—one a very definite vertigo and the other in which the patient feels merely a sensation of unsteadiness or possibly faintness. One may classify the cases into (1) a definite cerebral lesion, (2) wrong impressions coming up to a healthy brain, (3) defect in the blood-supply of the brain, (4) poisons, (5) stomach troubles, (6) neurasthenia. A case of brain-tumour will almost always present other features than giddiness, and if this be a prominent symptom the tumour is very likely in the cerebellum. Epilepsy as a cause of giddiness is often a difficult cause to determine ; if this be the only sign characteristic features may be recurrences and loss of consciousness. Wrong impressions reaching the brain and causing giddiness may come from the ear and eye. The ear lesion is generally in the internal ear—either hæmorrhage, syphilis, or chronic sclerosis, and the patient is also deaf. If in the eye the defect is a paresis of one or more of the eye-muscles, either external or internal. Giddiness from vascular troubles is a confusion of consciousness and not a strict vertigo ; it is especially marked on stooping to do ordinary things, *e.g.*, tying a bootlace. It is probably caused by a defect in the elasticity of the blood-vessels and, consequently, of the regulation of the blood-supply. Giddiness from gastric disturbances is generally due to flatulence. Tobacco is very apt to cause giddiness, partly owing to a specific effect of the poison on the nerve-cells, partly to a diminished capillary pressure.

**Chronic Headache.**—Hinshelwood[6] is of opinion that probably 50 per cent. of cases of chronic headache are due to eye strain—a cause which is often overlooked, because there is often no pain in the eyes but only headache. Practically, when headache is induced by the use of the eyes, it is of ocular origin, the patient is free in the morning, and the headache begins with the day's work. The commonest causes are astigmatism and hypermetropia, it is rarely due to myopia. The refractive error is generally a slight one, seldom a high one. When the error is a high one the patient abandons all effort to improve his vision by contracting the ciliary muscle, and so does not get headache. Next to eye-strain, the commonest cause of headache is some toxæmia which should be suspected when there is headache in the morning on waking. Dyspepsia, rheumatism, or gout may be the cause, but the most important is constipation. The pain is usually occipital or vertical, but may be frontal. The headache may be due to syphilis, and then it is usually nocturnal in time. Other causes are Bright's disease and disease of the brain—hence in such cases the urine and the fundus oculi should always be examined.

[4] Med. Record, Feb. 20. [5] Clin. Jour., March 2. [6] Treatment, March. [7] Ibid.

(*To be continued.*)

